# Supplementary figures and images for: ECCsplorer: a pipeline to detect extrachromosomal circular DNA (eccDNA) from next-generation sequencing data
Source: BMC Bioinformatics. 2022 Jan 14;23:40. doi: 10.1186/s12859-021-04545-2 (PMC8760651; doi:10.1186/s12859-021-04545-2)

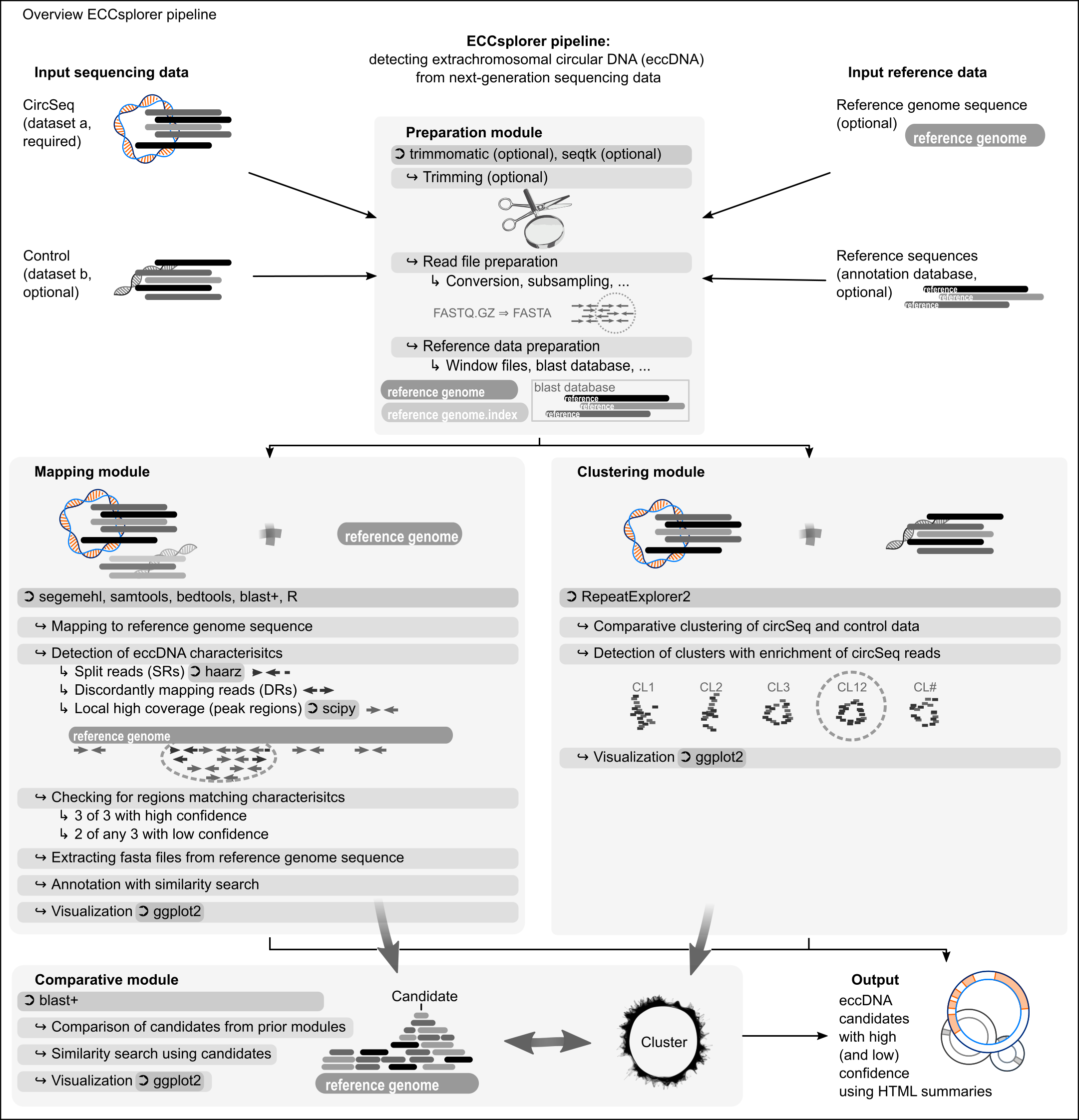

Supplement: Supplementary file 1 — Additional file 1. Figure S1: Extended version of Figure 1a with detailed pipeline overview. Our pipeline consists of four main modules: one for the data preparation module and three for the analysis modules. The execution of the different modules depends on the given input data. [file 12859_2021_4545_MOESM1_ESM.png]
