# Supplementary material for: ECCsplorer: a pipeline to detect extrachromosomal circular DNA (eccDNA) from next-generation sequencing data
Source: BMC Bioinformatics. 2022 Jan 14;23:40. doi: 10.1186/s12859-021-04545-2 (PMC8760651; doi:10.1186/s12859-021-04545-2)
Supplement: Supplementary file 2 — Additional file 2. Methods: Detailed methods for data generation (including circSeq), analysis using the ECCsplorer pipeline (including specific commands), comparison with other tools, hardware requirements, and a detailed description of each ECCsplorer pipeline modules. [file 12859_2021_4545_MOESM2_ESM.docx]

**ECCsplorer: a pipeline to detect extrachromosomal circular DNA (eccDNA) from next-generation sequencing data**

# Supplementary Methods

## Plant material and DNA extraction

*Beta vulgaris ssp. vulgaris* genotype KWS2320 was grown under greenhouse conditions by KWS Saat SE & Co. KGaA. Freshly harvested inflorescences were shipped on ice, stored at 4 °C and whole genomic DNA was extracted within a week. For one pooled plant sample, whole genomic DNA was extracted using the plant DNeasy mini kit (Qiagen) according to the manufacturer's instructions.

## Extrachromosomal circular DNA enrichment

For the removal of large genomic linear fragments 3 µg of genomic DNA was purified using a QIAquick PCR purification kit (Qiagen) according to the manufacturer's instructions. Remaining linear DNA from 25 µl of the purification product was further removed using the Plasmid-Safe^TM^ ATP-dependent DNase (Epicentre) exonuclease according to the manufacturer's instructions, except that the incubation at 37 °C was elongated to 17 h. The DNA sample was cleaned up using a standard ethanol/glycogen precipitation protocol by adding 0.1 volume of sodium acetate (3M), 2.5 volume ethanol and 1 µl glycogen (Beckman Coulter). The precipitated circular DNA was enriched by random rolling circle amplification (rRCA) using the Illustra^TM^ TempliPhi^TM^ amplification kit (GE Healthcare). Therefore, the DNA pellet from the precipitation was directly resuspended in 5 µl of the TempliPhi^TM^ sample buffer. The reaction was performed according to the manufacturer's instructions, except that the incubation at 28 °C was performed for 65 h. For the determination of the DNA concentration, 1 µl of the enriched DNA sample was purified using the GeneJet DNA clean up kit (Thermo Fisher Scientific) according to the manufacturer's instructions. Then, the DNA concentration was measured optically using a NanoDrop^TM^ photometer (Thermo Fisher Scientific). The remaining sample was diluted to 1 ng/µl for library preparation and sequencing.

## Library preparation and sequencing

Library preparation using the Nextera^TM^ library kit and sequencing was performed through the commercial provider Macrogen (Macrogen Inc., Seoul, Korea, www.macrogen.com). Sequencing was performed using the HiSeq X Ten platform (Illumina) targeting 20 Gb of 2 x 250 nucleotide paired-end reads. A mapping to the reference genome sequence EL10 [1] using bowtie2 [2] with default settings revealed an average insert size of around 650 bp.

## Data analysis and running of the ECCsplorer pipeline

In order to illustrate and test the ECCsplorer’s operating principle, we created semi-artificial test datasets, available on https://github.com/crimBubble/ECCsplorer/tree/master/testdata. These contain a region (0.6 Mb) from the *B. vulgaris* reference genome sequence as the control dataset. To simulate retrotransposon enrichment typical for some eccDNAs, we added multiple concatenated *Beetle7* copies with LTR/LTR and solo-LTR junctions to obtain circSeq data. For the creation of the artificial circSeq and the control datasets (25,000 spots each with 2 x 200 bp) we ran dwgsim (https://github.com/nh13/DWGSIM) with default settings. The ECCsplorer pipeline was run with the trimming option enabled and the read count option set to auto.

$ python3 ECCsplorer.py aDNA_R1.fastq aDNA_R2.fastq gDNA_R1.fastq gDNA_R2.fastq -trm tru2 -d RefSeq_DB.fasta -ref RefGenSeq.fasta -cnt 3000 –dsa circsim –dsb control

For the validation of the ECCsplorer’s functionality, we profited from some of the first eccDNA enrichment studies available [3, 4]. CircSeq data from *A. thaliana* (ERR1830501 and corresponding control datasets ERR1830499), and similar data from *H. sapiens* muscle tissue (SRR6315430) were used, respectively. As references for the mapping module, the current reference genome assemblies were used, namely the *A. thaliana* TAIR10 (The Arabidopsis Information Resource, http://www.arabidopsis.org) and the *H. sapiens* hg38 (UCSC Genome Browser, https://genome.ucsc.edu/). Additionally, the corresponding gene and mRNA databases were retrieved as annotation databases. For both datasets, the ECCsplorer pipeline was run with the trimming option enabled and for the *H. sapiens* dataset the window size parameter has been increased to 250 bp.

$ python3 ECCsplorer.py ERR1830501_1.fastq.gz ERR1830501_2.fastq.gz ERR1830499_1.fastq.gz ERR1830499_2.fastq.gz -trm nex -d Araport11_genes.201606.cds.fasta ATCOPIA93.fasta -ref TAIR10.fa -dsa epi12 -dsb cntrl

$ python3 ECCsplorer.py SRR6315430_1.fastq.gz SRR6315430_2.fastq.gz -trm tru2 -d GRCh38_rna.fna -ref hg38_chr16.fa –win 250

For the reference-free detection eccDNA-enriched *B. vulgaris* reads were used (ERR6004146), respectively. As control, WGA reads from the same genotype, used for generation of the published genome assembly [5] (SRR869631), were retrieved. The ECCsplorer was run with the trimming option enabled, read count set to auto (using a genome coverage of 0.1×) with an estimated genome size of 750 MB and with otherwise standard parameters.

$ python3 ECCsplorer.py ERR6004146_1.fastq.gz ERR6004146_2.fastq.gz SRR6315430_1.fastq.gz SRR869631_2.fastq.gz -trm nex –cnt auto –rgs 750000000

## Comparison between the CircleMap and the ECCsplorer softwares

To enable a software comparison between the ECCsplorer and Circle-Map [6], circSeq data from *A. thaliana* and *H. sapiens* were mapped against the corresponding reference genomes. To achieve a typical Circle-Map output, the provided tutorial instructions for mapping and the identification of circular DNAs were followed (https://github.com/iprada/Circle-Map/wiki/Tutorial:-Identification-of-circular-DNA-using-Circle-Map-Realign).

BED files were retrieved from the outputs of the ECCsplorer and the Circle-Map tools, and from the provided material of the original studies [3, 4]. These were compared using BEDtools intersect (-u option) [7]. The results have been visualized using the R packages ggplot2 [8] (https://ggplot2.tidyverse.org/) and circlize [9] (https://jokergoo.github.io/circlize/).

## Hardware and required software

All ECCsplorer pipeline runs were performed on a Unix machine (Ubuntu 16.04 LTS, i7 6-gen with 64 GB). The ECCsplorer pipeline is implemented in Python 3 (3.5 or higher) with Biopython [10], Scipy [11] and pyRserve [12] and R [13] with ggplot2 [8], ggrepel [14], gridExtra [15] and dplyr [16] and 3^rd^ party tools Blast+ [17], Trimmomatic [18] (optional but recommended), seqtk [19] (optional but recommended for better performance), segemehl [20], SAMtools [21], BEDtools [7], RepeatExplorer2 [22, 23].

## Detailed description of pipeline modules

### ECCsplorer.py: pipeline coordination

The ECCsplorer.py coordinates the pipeline run. The command line functions are set up using the python package argparser. When started, the module performs the following steps:

1. Collect user command line input
2. Setup logging
3. Check tool availability and start the Rserve connection
4. Check user input (file types and necessary input)
5. Setup reference genome sequence and blast databases
6. Run preparation module
7. Run mapping module
8. Run clustering module
9. Run comparative module

### config.py: pipeline configuration

The config.py contains the configuration for the ECCsplorer pipeline including names of directories, PATH values, commands and set parameters of 3rd party bioinformatic tools and other analysis parameters.

In case to run the ECCsplorer pipeline properly you might edit the PATH values of several 3rd party tool. The default values are: TOOL_PATH = 'tool'

Example (segemehl): SEGEMEHL_PATH = 'segemehl.x'

If you did not add the segemehl tool to the $PATH environment variable you need to change this value to look like:

segemehl_PATH = '/directory/path/to/segemehl-0.3.4/segemehl.x'

The 3rd party tool parameters and the pipeline parameters might be edited to fit individual needs. The following parameters can be edited in the configuration file:

1. 3rd party tool parameters (segemehl, Blast+, RepeatExplorer2, Trimmomatic). For detailed information please refer to the corresponding manual.
2. Pipeline parameters (to be edited to fit individual needs):
3. Approximate eccDNA length: MAX_eccDNA_LENGTH & MIN_eccDNA_LENGTH
4. Merging distance of two candidates: MERGE_CLOSE_REGIONS
5. Peak finder parameters: PEAK_THRESHOLD & PEAK_DISTANCE
6. Enrichment threshold for high confidant eccDNA candidate regions: ENRICH_THRESHOLD
7. Reference sequence window size (default value): WINDOW_SIZE (-win/--window_size)
8. Minimal cluster proportion for cluster candidates: REPEX_ECC_PROPORTION
9. Approximate genome size in bp: REPEX_GENOME_SIZE
10. Samflags for discordant mapping reads: SAMFLAGS_DR & SAMFLAGS_DR_not
11. Image parameters: IMAGE_RES, IMAGE_WIDTH, IMAGE_HEIGHT & IMAGE_POINTS
12. Rserve port: PORT

Other pipeline parameters should not be edited.

### eccPrepare.py: preparation module

The eccPrepare.py prepares read files (trimming, converting, sub sampling, concatenating, etc.) for the following modules.

| Input: | User given read files |
| --- | --- |
| Applicable options: | -trm/--trim_reads; -cnt/--read_count; -m/--mode |
| Output: | 2 or 4 prepared FASTA read files and REPEATEXPLORER_READY.fa |

The read files are trimmed using the trimmomatic tool, which also enables the usage of compressed input. After trimming, the reads are converted (in parallel) to FASTA format using the SeqIO package (from biopython). If input files are already in FASTA format, both steps are skipped. The module part returns the current read files as paired files (dictionary) and single file names (list).

The current read files are further processed depending on user input. If the input consists four files or the PRExer option is set the following steps are performed:

1. Read checkup I/II (calculate optimal read length)
2. Explanation: For read clustering all input reads should be equal in length.
3. Optimal read length for minimal data loss is calculated for each read file (reads shorter than optimal length are discarded, reads longer are cut at the end).
4. Optimal read length is calculated using Scipy optimize.
5. Chosen optimal read length is minimal optimal read length overall.
6. Read checkup II/II (find maximal usable read count with optimal read length)
7. Explanation: For read clustering an equal number of reads from all data sets should be provided for comparative analysis.
8. Count read pairs from each data set which satisfy the optimal read length.
9. Chosen read count is lowest number of overall read count (no count option set), user set count (count option set as integer) or 0.1x genome coverage (count option set to auto).
10. Reads are prepared (with optimal read length and read count)
11. Prefix and suffix are added to the read identifiers for comparative analysis:
12. >PREFIX_read.id_#0/1 (forward read) and >PREFIX_read.id_#0/2 (reverse read).
13. Reads are sub-sampled to chosen read count.
14. Reads are interlaced (forward and reverse pairs are grouped together).
15. Reads are concatenated (data sets A and B are grouped together).
16. REPEATEXPLORER_READY.fa file is written.

### eccMapper.py: mapping module

The eccMapper.py coordinates the read mapping with segemehl. Resulting mappings are further analyzed and highly covered regions, split reads and discordant mapping reads are detected using peak.finder from scipy, haarz from segemehl and samtools (analysis of SAM-flags).

| Input: | 2 or 4 prepared FASTA read files |
| --- | --- |
| Applicable options: | -ref/--reference_genome; -d/--database; -win/--window_size; -dsa/--preA and -dsb/--preB |
| Output: | Mapping files, coverage files, mapping eccDNA candidates |

Within the module the following steps are performed:

1. Setup of reference related files.
2. Segmehl index file.
3. Read mapping using segemehl.
4. Alignment with segemehl algorithm
5. Collection of split reads (segemehl --splits option).
6. Collection of discordant mapping reads using SAM flags (2, 83, 163) with 2: reads not mapped in proper pair (samtools view -G) and 83/163: reads mapped with unusual orientation (rev-for) (samtools view -f)
7. Conversion from SAM format to BED format.
8. Creation of statistics information files.
9. Analysis of split reads (SR) to detect SR related regions on reference genome sequence.
10. SR analysis with haarz algorithm.
11. Merging of haarz detected regions (bedtools merge).
12. Detection of candidate regions on the reference genome sequence.
13. Creation of coverage files with bedtools coverage -mean over reference sequence windows (-win/--window_size option) for general coverage (map.all), SR coverage (map.SR) and discordant mapping reads (map.DR).
14. Detection of highly covered regions using peak.finder from scipy.
15. Write sorted peak region files (unsorted in temp) for general coverage (map.all), SR coverage (map.SR) and discordant mapping reads (map.DR).
16. Compare criteria to detect candidate regions. Regions are considered low confidant eccDNA candidate regions (lcECR) if at least 2 of 3 criteria are matched and high confidant eccDNA candidate regions (hcECR) if 3 of 3 are matched. Criteria are SR, DR and general high coverage.
17. bedtools intersect is used to find regions using PREa_regions-SR.bed, PREa_peak-region-all.bed and PREa_peak-region-DR.bed files.
18. Write hcECR file, and write and merge lcECR files.
19. Normalization of coverage. Coverage point is normalized to mapped base pairs (in window) per million mapped base pairs (overall) (BPM). Calculation of BPM is analogously performed to more the commonly known reads per million reads (RPM). But RPM is not appropriate in case of different read length in two data sets. Normalization is calculated in R. Normalized coverage = Raw coverage ⋅ 1,000,000 / Total mapped bases per dataset
20. Gathering mean coverage over hcECR and calculating enrichment scores. If no hcECR found, lcECR will be used for all following steps
21. Create coverage files and normalize.
22. Calculating coverage per base pair over enriched hcECR.
23. Extract enriched hcECR sequences from the reference genome sequence.
24. Blast enriched hcECR sequences.
25. Visualization of normalized coverage over the reference genome sequence. Using ggplot2 and saved as image (-img/--image_format option).
26. Normalization and visualization of enriched hcECR coverage.
27. Write a summary of mapping results (HTML).

### eccClusterer.py: clustering module

The eccClusterer.py coordinates the read clustering with RepeatExplorer2. Resulting clusters are further analyzed and unusual distributed clusters are detected.

| Input: | REPEATEXPLORER_READY.fa |
| --- | --- |
| Applicable options: | -cnt/--read_count; -tax/--taxon; -dsa/--preA and -dsb/--preB |
| Output: | Clustering files, clustering eccDNA candidates tables |

Within the module following steps are performed:

1. Clustering with RepeatExplorer2.
2. Rearranging and merging clustering results.
3. Detection of eccDNA cadidate clusters (ECCL)
4. Detection criteria are the data set read proportions of a cluster.
5. If the treatment data produces a read count proportion above the threshold (config.py: pipeline configuration page 1, default = 0.8 (80%)) the cluster is marked as ECCL.
6. Visualization of cluster abundance values.
7. Write a summary of clustering results (HTML).

### eccComparer.py: comparative module

The eccComparer.py coordinates the comparison of hcECR with ECCL. Resulting matches are summarized.

| Input: | Mapping eccDNA candidates (sequence), eccDNA candidate clusters |
| --- | --- |
| Applicable options: | – |
| Output: | Comparative analysis files, final eccDNA candidates |

Within the module following steps are performed:

1. Create a Blast+ database from hcECR sequences.
2. Blasting ECCL consensus sequences against hcECR database.
3. Summarize matches.
4. For each hcECR corresponding ECCL are merged.
5. Usually one hcECR corresponds to multiple ECCL which belong to fewer super clusters.
6. Visualization of matches and overview data.
7. Write a summary of comparative results (HTML).

# Additional references (methods)

1. Funk A, Galewski P, McGrath JM. Nucleotide-binding resistance gene signatures in sugar beet, insights from a new reference genome. The Plant Journal. 2018. doi:10.1111/tpj.13977.

2. Langmead B, Salzberg SL. Fast gapped-read alignment with Bowtie 2. Nature Methods. 2012;9:357–9.

3. Lanciano S, Carpentier M-C, Llauro C, Jobet E, Robakowska-Hyzorek D, Lasserre E, et al. Sequencing the extrachromosomal circular mobilome reveals retrotransposon activity in plants. PLoS Genetics. 2017;13:e1006630–50.

4. Møller HD, Mohiyuddin M, Prada-Luengo I, Sailani MR, Halling JF, Plomgaard P, et al. Circular DNA elements of chromosomal origin are common in healthy human somatic tissue. Nat Commun. 2018;9:1069–81.

5. Dohm JC, Minoche AE, Holtgräwe D, Capella-Gutiérrez S, Zakrzewski F, Tafer H, et al. The genome of the recently domesticated crop plant sugar beet (*Beta vulgaris*). Nature. 2014;505:546–9.

6. Prada-Luengo I, Krogh A, Maretty L, Regenberg B. Sensitive detection of circular DNAs at single-nucleotide resolution using guided realignment of partially aligned reads. BMC Bioinformatics. 2019;20:663.

7. Quinlan AR. BEDTools: The Swiss-Army Tool for Genome Feature Analysis. Curr Protoc Bioinformatics. 2014;47:11.12.1-34.

8. Wickham H. ggplot2: Elegant Graphics for Data Analysis. Springer; 2016.

9. Gu Z, Gu L, Eils R, Schlesner M, Brors B. circlize implements and enhances circular visualization in R. Bioinformatics. 2014;30:2811–2.

10. Cock PJA, Antao T, Chang JT, Chapman BA, Cox CJ, Dalke A, et al. Biopython: freely available Python tools for computational molecular biology and bioinformatics. Bioinformatics. 2009;25:1422–3.

11. Virtanen P, Gommers R, Oliphant TE, Haberland M, Reddy T, Cournapeau D, et al. SciPy 1.0: fundamental algorithms for scientific computing in Python. Nature Methods. 2020;17:261–72.

12. Heinkel R. pyRserve: A Python client to remotely access the R statistic package via network. Python. 2017. https://pypi.org/project/pyRserve.

13. R Core Team. R: A language and environment for statistical computing. Vienna, Austria: R Foundation forStatistical Computing; 2013. https://www.R-project.org/.

14. Slowikowski K, Schep A, Hughes S, Lukauskas S, Irisson J, Kamvar Z. ggrepel: automatically position non-overlapping text labels with ‘ggplot2.’ R. 2019. https://CRAN.R-project.org/package=ggrepel.

15. Auguie B, Antonov A. gridExtra: miscellaneous functions for “grid” graphics. R. 2017. https://CRAN.R-project.org/package=gridExtra.

16. Wickham H, Francois R, Henry L, Müller K. dplyr: a grammar of data manipulaton. R package version 0.8. 0.1. R. 2019. https://CRAN.R-project.org/package=dplyr.

17. Camacho C, Coulouris G, Avagyan V, Ma N, Papadopoulos J, Bealer K, et al. BLAST+: architecture and applications. BMC Bioinformatics. 2009;10:421.

18. Bolger AM, Lohse M, Usadel B. Trimmomatic: a flexible trimmer for Illumina sequence data. Bioinformatics. 2014;30:2114–20.

19. Li H, Bufallo V, Murray K, Langhorst B, Klötzl F, Jain C. Seqtk: a fast and lightweight tool for processing FASTA or FASTQ sequences. 2013. https://github.com/lh3/seqtk.

20. Hoffmann S, Otto C, Doose G, Tanzer A, Langenberger D, Christ S, et al. A multi-split mapping algorithm for circular RNA, splicing, trans-splicing and fusion detection. Genome Biology. 2014;15:R34–50.

21. Li H, Handsaker B, Wysoker A, Fennell T, Ruan J, Homer N, et al. The sequence alignment/map format and SAMtools. Bioinformatics. 2009;25:2078–9.

22. Novák P, Neumann P, Macas J. Graph-based clustering and characterization of repetitive sequences in next-generation sequencing data. BMC Bioinformatics. 2010;11:378–90.

23. Novák P, Neumann P, Macas J. Global analysis of repetitive DNA from unassembled sequence reads using RepeatExplorer2. Nature Protocols. 2020;15:3745–76.
