# Supplementary material for: ECCsplorer: a pipeline to detect extrachromosomal circular DNA (eccDNA) from next-generation sequencing data
Source: BMC Bioinformatics. 2022 Jan 14;23:40. doi: 10.1186/s12859-021-04545-2 (PMC8760651; doi:10.1186/s12859-021-04545-2)
Supplement: Supplementary file 3 — Additional file 3. Tables S1–S9: Results from eccDNA detection using the ECCsplorer pipeline. (1) Case study I: Top results from mapping module. (2) Case study I: Top results from clustering module. (3) Case study I: Top results from comparative module. (4) Case study II: Top results from mapping module. (5) Case study II: Top results from clustering module. (6) Case study II: Top results from comparative module. (7) Case study II: Comparison of tools. (8) Case study III: Top results from mapping module. (9) Case study IV: Top results from clustering module. [file 12859_2021_4545_MOESM3_ESM.pdf]

Supplementary Table 1 (semi-artificial data, `running mode: all > mapping module`)

Supplementary Table 1: Top results from mapping module using test data (circsim and control). Using this dataset, the one artificially created eccDNA candidate was detected.

Candidate	Enrichment score	Length [bp]	Position	Best hit annotation
eccCand_001	46.5	6695	chr3:86641-93336	Beetle-7#LTR/Gypsy/chromovirus/CRM@Beta_vulgaris

Supplementary Table 2 (semi-artificial data, `running mode: all > clustering module`)

Supplementary Table 2: Top results from clustering module using test data (circsim and control). Using this dataset, the one artificially created eccDNA candidate was detected as one supercluster.

Cluster	Supercluster	Size [reads]	epiRIL proportion	Best hit annotation
CL1	1	401	0.97	All/repeat/mobile_element/Class_I/LTR/Ty3_gypsy/chromovirus/CRM
CL2	1	370	0.97	All/repeat/mobile_element/Class_I/LTR/Ty3_gypsy/chromovirus/CRM
CL3	1	330	0.979	All/repeat/mobile_element/Class_I/LTR/Ty3_gypsy/chromovirus/CRM
CL4	1	321	0.984	All/repeat/mobile_element/Class_I/LTR/Ty3_gypsy/chromovirus/CRM
CL5	1	291	0.973	All/repeat/mobile_element/Class_I/LTR/Ty3_gypsy/chromovirus/CRM
CL6	1	280	0.979	All/repeat/mobile_element/Class_I/LTR/Ty3_gypsy/chromovirus/CRM
CL7	1	260	0.992	All/repeat/mobile_element/Class_I/LTR/Ty3_gypsy/chromovirus/CRM
CL8	1	254	0.992	All/repeat/mobile_element/Class_I/LTR/Ty3_gypsy/chromovirus/CRM
CL9	1	215	0.967	All/repeat/mobile_element/Class_I/LTR/Ty3_gypsy/chromovirus/CRM
CL10	1	209	0.981	All/repeat/mobile_element/Class_I/LTR/Ty3_gypsy/chromovirus/CRM
CL11	1	206	0.981	All/repeat/mobile_element/Class_I/LTR/Ty3_gypsy/chromovirus/CRM
CL12	1	172	0.977	All/repeat/mobile_element/Class_I/LTR/Ty3_gypsy/chromovirus/CRM
CL13	1	166	0.976	All/repeat/mobile_element/Class_I/LTR/Ty3_gypsy/chromovirus/CRM

Supplementary Table 3 (semi-artificial data, `running mode: all > comparative module`)

Supplementary Table 3: Top results from comparative module using test data (circsim and control). Using this dataset, the one artificially created eccDNA candidate was detected.

Candidate	Enrichment score	Best hit annotation (mapping module)	Associated clusters	epiRIL proportion (per cluster)	Best hit annotation (clustering module)
eccCand_001	46.5	Beetle-7#LTR/Gypsy/chromovirus/CRM@Beta_vulgaris	CL1	0.97	All/repeat/mobile_element/Class_I/LTR/Ty3_gypsy/chromovirus/CRM
			CL2	0.97	
			CL3	0.979	
			CL4	0.984	
			CL5	0.973	
			CL6	0.979	
			CL7	0.992	
			CL8	0.992	
			CL9	0.967	
			CL10	0.981	
			CL11	0.981	
			CL12	0.977	
			CL13	0.976	

Supplementary Table 4 (*A. thaliana* data, `running mode: all > mapping module`)

Supplementary Table 4: Top results from mapping module using *A. thaliana* data (epi12 and WT). Using this data set, 13 eccDNA candidate regions were detected.

Candidate	Enrichment score	Length [bp]	Position	Best hit annotation
eccCand_001	56	5332	chr5:5629975-5635307	EVD AT5TE20395|+|5629978|5635310|ATCOPIA93|LTR/Copia|5333
eccCand_002	28	6649	chr1:12753744-12760393	EVD AT5TE20395|+|5629978|5635310|ATCOPIA93|LTR/Copia|5333
eccCand_003	12	424	chr5:11813591-11814015	
eccCand_004	10	127	chr3:10182070-10182197	EVD AT5TE20395|+|5629978|5635310|ATCOPIA93|LTR/Copia|5333
eccCand_005	7	744	chr2:5829018-5829762	AT2G13895.1
eccCand_006	4.5	8863	chr2:3500250-3509113	150_gi|26556996|ref|NC_001284.2|#organelle/mitochondria
eccCand_007	2	34116	chr2:3346113-3380229	150_gi|26556996|ref|NC_001284.2|#organelle/mitochondria
eccCand_008	2	12029	chr5:11838404-11850433	
eccCand_009	1.33	2971	chr2:1622-4593	23_gi|604854740|gb|KJ507198.1|#45S_rDNA/18S_rDNA
eccCand_010	1.33	943	chr2:2937853-2938796	443_gi|7525012|ref|NC_000932.1|#organelle/plastid
eccCand_011	1	3446	chr2:6785-10231	24_25S_PS_CL2Contig6_rc/3985-7377#45S_rDNA/25S_rDNA
eccCand_012	1	4015	chr2:3268139-3272154	150_gi|26556996|ref|NC_001284.2|#organelle/mitochondria
eccCand_013	1	25814	chr3:14191918-14217732	24_25S_PS_CL2Contig6_rc/3985-7377#45S_rDNA/25S_rDNA

Supplementary Table 5 (*A. thaliana* data, `running mode: all > clustering module`)

Supplementary Table 5: Top 20 results from clustering module using *A. thaliana* data (epi12 and WT). Using this data set, multiple eccDNA candidate regions were detected by the clustering approach.

Cluster	Super cluster	Size [reads]	epiRIL proportion	Best hit annotation
CL1	2	4060	1.0	All/repeat/mobile_element/Class_I/LTR/Ty1_copia/Ale
CL2	3	3373	0.857	All/organelle/mitochondria
CL6	3	2530	0.804	All/organelle/mitochondria
CL10	10	2362	1.0	All
CL14	2	2212	0.999	All/repeat/mobile_element/Class_I/LTR/Ty1_copia/Ale
CL16	2	1923	1.0	All/repeat/mobile_element/Class_I/LTR/Ty1_copia/Ale
CL17	8	1796	1.0	All
CL18	3	1629	0.878	All/organelle/mitochondria
CL19	9	1480	1.0	All
CL23	14	1345	1.0	All
CL26	8	1303	1.0	All
CL32	18	1138	0.998	All
CL33	9	1129	1.0	All
CL37	20	943	1.0	All
CL42	23	903	0.998	All
CL49	28	750	1.0	All
CL56	35	628	0.994	All/repeat
CL61	39	572	1.0	All
CL63	2	544	0.996	All/repeat/mobile_element/Class_I/LTR/Ty1_copia/Ale
CL78	53	401	1.0	All

Supplementary Table 6 (*A. thaliana* data, `running mode: all > comparative module`)

Supplementary Table 6: Top results from comparative module using *A. thaliana* data (epi12 and WT). Using this data set, eight eccDNA candidate regions were detected across both approaches (mapping and clustering) and are considered eccDNA candidates with high confidence.

<table>
<thead>
<tr><th>Candidate</th><th>Enrichment score</th><th>Best hit annotation
(mapping module)</th><th>Associated clusters</th><th>epiRIL proportion
(per cluster)</th><th>Best hit annotation
(clustering module)</th></tr>
</thead>
<tbody>
<tr><td>eccCand_001</td><td>56</td><td>EVD AT5TE20395|+|5629978|5635310|
ATCOPIA93|LTR/Copia|5333</td><td>CL1
CL14
CL16
CL63</td><td>1.0
0.999
1.0
0.996</td><td>All/repeat/mobile_element/
Class_I/LTR/Ty1_copia/Ale</td></tr>
<tr><td>eccCand_002</td><td>28</td><td>EVD AT5TE20395|+|5629978|5635310|
ATCOPIA93|LTR/Copia|5333</td><td>CL1
CL14
CL16
CL63</td><td>1.0
0.999
1.0
0.996</td><td>All/repeat/mobile_element/
Class_I/LTR/Ty1_copia/Ale</td></tr>
<tr><td>eccCand_004</td><td>10</td><td>EVD AT5TE20395|+|5629978|5635310|
ATCOPIA93|LTR/Copia|5333</td><td>CL1</td><td>1.0</td><td>All/repeat/mobile_element/
Class_I/LTR/Ty1_copia/Ale</td></tr>
<tr><td>eccCand_006</td><td>4.5</td><td>150_gi|26556996|ref|NC_001284.2|
#organelle/mitochondria</td><td>CL2
CL6
CL18
CL415</td><td>0.857
0.804
0.878
0.926</td><td>All/organelle/mitochondria</td></tr>
<tr><td>eccCand_007</td><td>2</td><td>150_gi|26556996|ref|NC_001284.2|
#organelle/mitochondria</td><td>CL2
CL6
CL18
CL415</td><td>0.857
0.804
0.878
0.926</td><td>All/organelle/mitochondria</td></tr>
<tr><td>eccCand_011</td><td>1</td><td>24_25S_PS_CL2Contig6_rc/3985-7377
#45S_rDNA/25S_rDNA</td><td>CL288
CL405</td><td>0.956
0.862</td><td>All/repeat/rDNA/45S_rDNA/
25S_rDNA</td></tr>
<tr><td>eccCand_012</td><td>1</td><td>150_gi|26556996|ref|NC_001284.2|
#organelle/mitochondria</td><td>CL89</td><td>0.825</td><td>All</td></tr>
<tr><td>eccCand_013</td><td>1</td><td>24_25S_PS_CL2Contig6_rc/3985-7377
#45S_rDNA/25S_rDNA</td><td>CL288
CL405</td><td>0.956
0.862</td><td>All/repeat/rDNA/45S_rDNA/
25S_rDNA</td></tr>
</tbody>
</table>

Supplementary Table 7 (*A. thaliana* data, Comparison of ECCsplorer, Circle-Map, and originally published candidates)

Supplementary Table 7: Overlapping candidate regions using *A. thaliana* data (epi12 and WT) from each mapping analysis (**ECC**splorer pipeline, **C**ircle-**M**ap, and originally published data by **Lan**ciano et al. (2017)) examined using BEDtools.

Chromosome	Start	End	Quality	Method	Chromosome	Start	End	Quality	Method
chr1	12753744	12760393	hconf	ECC	chr1	12754300	12760400	hconf	Lan
chr2	3268139	3272154	hconf	ECC	chr2	3268140	3270259	---	CM
chr2	3268139	3272154	hconf	ECC	chr2	3270663	3273277	---	CM
chr2	3346113	3380229	hconf	ECC	chr2	3344596	3346513	---	CM
chr2	3346113	3380229	hconf	ECC	chr2	3349795	3350330	---	CM
chr2	3346113	3380229	hconf	ECC	chr2	3350818	3353174	---	CM
chr2	3346113	3380229	hconf	ECC	chr2	3355398	3356724	---	CM
chr2	3346113	3380229	hconf	ECC	chr2	3358494	3359166	---	CM
chr2	3346113	3380229	hconf	ECC	chr2	3359287	3378942	---	CM
chr2	3500250	3509113	hconf	ECC	chr2	3499864	3501375	---	CM
chr3	10182070	10182197	hconf	ECC	chr3	10181900	10182400	hconf	Lan
chr5	5629975	5635307	hconf	ECC	chr5	5630000	5635300	hconf	Lan
chr1	12362558	12363626	---	CM	chr1	12362700	12363200	hconf	Lan
chr1	16582926	16583270	---	CM	chr1	16583000	16583200	hconf	Lan
chr2	5829019	5829758	---	CM	chr2	5829200	5829400	hconf	Lan
chr3	11357284	11358090	---	CM	chr3	11357300	11357900	hconf	Lan
chr4	4594263	4594725	---	CM	chr4	4594400	4594600	hconf	Lan

Supplementary Table 8 (*H. sapiens* data, `running mode: map > mapping module`)

Supplementary Table 8: Top results from mapping module using *H. sapiens* data (circSeq). Only one eccDNA candidate region was detected with high confidence.

Additionally, 841 eccDNA candidate region were detected with low confidence.

Candidate	Enrichment score	Length [bp]	Position	Best hit annotation
eccCand_001	0.16	22722	chr16:87568894-87591666	MFN1 mitofusin 1 [NM_033540.3]

Supplementary Table 9 (*B. vulgaris* data, `running mode: clu > clustering module`)

Supplementary Table 9: Top results from clustering module using *B. vulgaris* data (circSeq and WGA). Cluster contigs were annotated by a nucleotide similarity search (online BLAST) against the NCBI database with default settings (organism ~ viridiplantae).

Cluster	Super cluster	Size [reads]	epiRIL proportion	Best hit annotation	NCBI Blast best hit annotation
CL3	1	5593	0.996	All/organelle/mitochondria	mitochondrial minicircle a/d [X04983.1, X04984.1]
CL5	1	3510	0.998	All/organelle/mitochondria	mitochondrial minicircle d [X04984.1]
CL6	1	3292	0.994	All/organelle/mitochondria	mitochondrial minicircle a [X04983.1]
CL10	1	2691	0.989	All/organelle/mitochondria	mitochondrial minicircle pO [X00641.1]
CL11	1	2633	0.994	All/organelle/mitochondria	mitochondrial minicircle a [X04983.1]
CL13	1	2343	0.997	All/organelle/mitochondria	mitochondrial minicircle d [X04984.1]
CL15	1	2140	0.999	All/organelle/mitochondria	mitochondrial minicircle d [X04984.1]
CL16	1	2099	0.994	All/organelle/mitochondria	mitochondrial minicircle a [X04983.1]
CL17	1	1958	0.996	All/organelle/mitochondria	mitochondrial minicircle d [X04984.1]
CL18	1	1954	0.994	All/organelle/mitochondria	mitochondrial minicircle a [X04983.1]
CL21	1	1763	0.997	All/organelle/mitochondria	mitochondrial minicircle d [X04984.1]
CL38	1	816	0.987	All/organelle/mitochondria	mitochondrial minicircle d/pO [X04984.1, X00641.1]
